# Influenza A virus infects pulmonary microvascular endothelial cells leading to microvascular leakage and release of pro-inflammatory cytokines

**DOI:** 10.7717/peerj.11892

**Published:** 2021-08-03

**Authors:** Tiantian Han, Yanni Lai, Yong Jiang, Xiaohong Liu, Danhua Li

**Affiliations:** 1 Guangzhou University of Chinese Medicine, Guangzhou, China; 2 Respiratory Department, Hospital of Integrated Traditional Chinese and Western Medicine, Shenzhen, Guangdong, China; 3 Respiratory Department, The First Affiliated Hospital of Guangzhou University of Chinese Medicine, Guangzhou, Guangdong, China; 4 Clinical Lab, The First Affiliated Hospital of Guangzhou University of Chinese Medicine, Guangzhou, Guangdong, China

**Keywords:** Influenza A, Pulmonary microvascular, Endothelial cells, Vascular leakage

## Abstract

**Objective:**

To investigate the replication of influenza A virus A/Puerto Rico/8/34 (H1N1) in pulmonary microvascular endothelial cells and its effect on endothelial barrier function.

**Methods:**

Human pulmonary microvascular endothelial cells were infected with influenza A/Puerto Rico/8/34 (H1N1) virus. Plaque reduction assay, real-time quantitative PCR, immunofluorescence staining, and western blot were used to elucidate the replication process of virus-infected endothelial cells. In addition, real-time quantitative PCR was used to detect the relative expression levels of mRNA of some inflammatory factors. The endothelial resistance assay was used to determine the permeability of the endothelial monolayer. Excavation and analysis of data from open databases, such as the GeneCards database, DAVID Bioinformatics Resources, STRING search tool, and DGIdb database determined the genes, proteins, and signal pathways related to microvascular leakage caused by the H1N1 virus, and predicted the drugs that could be effective for treatment.

**Results:**

*In vitro* experiments showed that the influenza virus can infect endothelial cells, leading to a significant increase in the permeability of pulmonary microvascular endothelial cells and the release of pro-inflammatory cytokines, but does not efficiently replicate in endothelial cells. A total of 107 disease-related target genes were obtained from the Gene-cards database. Kyoto Encyclopedia of Genes and Genomes (KEGG) enrichment analysis showed that these genes mainly affected the pathways related to “Inflammatory bowel disease” (IBD), “Chagas disease” (American trypanosomiasis), “Influenza A”, and also played a key role in anti-inflammation and regulation of immunity. After enrichment analysis, 46 hub genes were screened. A total of 42 FDA-approved drugs corresponding to the hub genes were screened from the DGIdb database, and these could be formulated for topical application. In addition, these drugs can be used to treat other diseases, including cancer, inflammatory diseases, immune system disorders, and cardiovascular diseases.

**Conclusion:**

H1N1 influenza virus affects the barrier function of endothelial cells indirectly. Combined with bioinformatics tools, we can better understand the possible mechanism of action of influenza A (H1N1) virus causing pulmonary microvascular leakage and provide new clues for the treatment of pulmonary microvascular leakage.

## Introduction

The spread of the influenza virus to the lower respiratory tract can cause viral pneumonia, which can then develop into acute lung injury and acute respiratory distress syndrome (ARDS). ARDS causes a marked increase in pulmonary microvascular leakage leading to pulmonary edema, hypoxemia, and respiratory failure (Daoud et al., 2019; Mauad et al., 2010). Recent data indicate that the influenza virus can directly or indirectly activate or damage lung endothelial cells, leading to increased microvascular leakage (Armstrong, Mubareka & Lee, 2013; Lobo et al., 2019). Therefore, the pulmonary microvascular endothelium can be used as a therapeutic target for severe influenza.

Previous studies have used umbilical vein endothelial cells or pulmonary microvascular endothelial cells to study the replication kinetics and host response of some influenza A virus strains (Sumikoshi et al., 2008). Other studies have revealed that the highly pathogenic avian influenza H7N9 influenza virus and the highly pathogenic H5N1 virus can effectively replicate in human lung microvascular endothelial cells, which may contribute to the viral pathogenesis and dissemination of the virus beyond the respiratory tract (Short, Kuiken & Van Riel, 2019; Zeng et al., 2012). However, the replication of the A/Puerto Rico/8/34 (H1N1) influenza virus in human lung microvascular endothelial cells and the effect of this strain on endothelial cells remain unclear.

In this study, we used human lung microvascular endothelial cells to understand the replication dynamics and effects of the influenza virus strain A/Puerto Rico/8/34 (H1N1) on pulmonary microvascular endothelial cells. We used bioinformatics tools to conduct text mining of biomedical literature, identify corresponding and targeted genes, proteins, and signal pathways from previous studies, and establish a variety of network relationships between drugs/molecules/targets and diseases. In addition, the the connection between the new genes and pathology was revealed. The computational prediction of multi-target drugs not only helped to discover more effective drugs but also provided great possibilities for studying new pharmacological targets and promoting drug repositioning and development in the pharmaceutical industry.

In this study, we demonstrated that, in *vitro*, Influenza A/Puerto Rico/8/34 (H1N1) virus can infect the pulmonary microvascular endothelium, and the infection can lead to the loss of barrier integrity. Finally, we used bioinformatics tools to explore the mechanism by which vascular leakage may increase, and predict some drugs that can reduce vascular leakage.

## Material and Methods

### Virus strains

Influenza A/Puerto Rico/8/34 (H1N1) virus was donated by the Guangzhou Institute of Respiratory Diseases. The virus was propagated in 9 days-old specific pathogen-free (SPF) embryonated hen eggs. Each chicken embryo was inoculated with 0.1ml of 100-fold diluted virus solution, and the hole was sealed with wax liquid. Following successful inoculation of the chicken embryos with the virus, they were incubated at 37 °C, for 48 h. The allantoic fluid was collected, centrifuged at 3000 rp for 5 min, to obtain the supernatant. The viral titer was detected by plaque assays (Furuta et al., 2002). The virus was packaged and stored at −80 °C.

### Cell culture and viral infection of cells

Madin-Darby canine kidney (MDCK) cell was a gift from the Institute of Respiratory Diseases of Guangzhou Medical University and the State Key Laboratory of Respiratory Diseases (Guangzhou, China). MDCK cells were maintained in Dulbecco’s modified Eagle’s medium (DMEM) supplemented with 10% fetal bovine serum, 100 U/ml penicillin, and 100 U/ml streptomycin at 37 °C under 5% CO_2_ (Lai et al., 2021). The human lung microvascular endothelial cell (HULEC-5a) was obtained from The Global Bioresource Center (ATCC CRL-3244). The HULEC-5a cells were maintained in Endothelial Cell Medium (ECM) at 37 °C under 5% CO_2_. The ECM contained basal medium, 5% fetal bovine serum, 100 U/ml penicillin, 100 U/ml streptomycin, and 1% Endothelial cell growth supplement (ECGS). Unless otherwise stated, and in the priming experiment, influenza virus was added to the MDCK cells and HULEC-5a cells in serum-free media at a multiplicity of infection (MOI) of 0.00001, 0.0001, 0.001, and 0.01 for two hours. The virus was removed, and DMEM containing 2 µg/ml tosylphenylalanyl chloride methyl ketone (TPCK) treated trypsin and 1% bovine serum albumin was added. All infections occurred within 48 hours (Li et al., 2011).

### Plaque formation assay

The experimental method of plaque reduction was based on a previous description (Furuta et al., 2002), as follows: MDCK cells (6 ×10^5^ cells/well) were seeded in 12-well plates and cultured at 37 °C under 5% CO_2_ for 24 h (Lai et al., 2021). After infection with influenza virus, MOI 0.01, MDCK, and HULEC-5a cells were incubated for 48 h. The cells were then washed with phosphate buffer saline (PBS) and the supernatants collected from the virus-infected HULEC-5a and MDCK cells. The supernatants were diluted 10^3^-fold, 10^4^-fold, 10^5^-fold, 10^6^-fold, and 10^7^-fold. The virus inoculums were removed, and the cells were washed three times with PBS. Next, the cell monolayers were overlaid with agar overlay medium (DMEM supplemented with 1% low melting point agarose and 2.5 µg/ml TPCK-treated trypsin) and incubated at 37 °C for 3 days. The cell monolayers were fixed with 4% paraformaldehyde for 2 h, the agarose overlays were then removed, and the cell monolayers were stained with 2%(w/v) crystal violet (Lai et al., 2021).

### Real-Time quantitative PCR analysis

Total cellular RNA was extracted using the Trizol reagent (Invitrogen, Carlsbad, CA, United States) according to the manufacturer’s instructions. RNA was reverse transcribed into cDNA using the reverse transcription kit (TaKaRa, Shiga, Japan). SYBR Green real-time PCR amplification and detection were performed using the ABI 7500 system (Applied Biosystems, Foster City, USA). The PCR system comprised of, 25 µl reaction buffer (RNA17 µl RNA template, 5 µl M-MLV 5 × reaction buffer, primer 50p molar buffer, 0.1mMd nucleotide, 25 units ribonuclease inhibitor and 200 units M-MLVRT polymerase). 18s rRNA was regarded as a house-keeping gene (Armstrong et al., 2012; Chunlian et al., 2014; Lai et al., 2020; Plotnikova, Klotchenko & Vasin, 2016; Yang et al., 2015).

IL-1β (forward primer) 5′-AGCTGATGGCCCTAAACAGA-3′

IL-1β (reverse primer) 5′-TGGTGGTCGGAGATTCGTAG-3′

TNF-α (forward primer) 5′-TGTAGCCCATGTTGTAGCAAA-3′

TNF-α (reverse primer) 5′-CAAAGTAGACCTGCCCAGACT-3′

NP (forward primer) 5′-GCACCAAACGGTCTTACGAA-3 ′

NP(reverse primer) 5′-TTTGGATCAACCGTCCCTCA-3 ′

18s rRNA(forward primer) 5′-GATGGAAAATACAGCCAGGTCCTA-3′

18s rRNA(forward primer) 5′-TTCTTCAGTCGCTCCAGGTCTT-3′

IL-4(forward primer) 5′-AGCTGATCCGATTCCTGAAAC-3 ′

IL-4 (reverse primer) 5′-AACGTACTCTGGTTGGCTTC-3 ′

IL-6(forward primer) 5′-CCACTCACCTCTTCAGAACG-3 ′

IL-6(forward primer) 5′-CATCTTTGGAAGGTTCAGGTTG-3 ′

### Immunofluorescence staining

HULEC-5a (1.5 ×10^5^ cells/well) and MDCK cells (1 ×10^5^ cells/well) were cultured on coverslips for 24 h. After infection with the influenza virus (MOI 0.01), they were incubated for 48 h. The cells were washed three times with phosphate buffer saline (PBS), fixed with 4% (v/v) paraformaldehyde in PBS for 15 min, and permeabilized with 0.5% Triton X-100 (prepared in PBS) permeate at room temperature for 20 minutes (Kim et al., 2015). For immunocytochemistry analysis of the H1N1 virus, the cells were incubated with mouse anti-NP protein antibody, followed by Alexa Fluor 488-conjugated anti-mouse IgG. The nucleus was fluorescently labeled with 4′,6-diamidino-2-phenylindole (DAPI; Sigma-Aldrich, St. Louis, MO) (Kim et al., 2015). Coverslips were mounted on the slide, with a mounting solution containing an anti-fluorescence quencher. Fluorescent images were obtained using a fluorescence microscope.

### Western blot

Lysates were prepared using the Radio Immunoprecipitation Assay (RIPA) lysis buffer and separated by 10% polyacrylamide gels. Proteins were transferred to nitrocellulose membranes, blocked for 1 h using 5% BSA, and probed overnight with a primary antibody at 4 °C. After washing, the blots were incubated with HRP-conjugated secondary antibodies for 1 h, washed, and visualized by advanced chemiluminescence (Amersham). Band intensity was quantified using Image J (NIH) and normalized to the loading control after background correction (Sugiyama et al., 2016). The NP antibody used to detect the influenza virus and the antibody to detect GAPDH were all purchased from GENE Tex (Southern California, Unites States). The goat anti-mouse IgG and goat anti-rabbit IgG were purchased from Jackson Immuno Research Laboratories Inc. (Pennsylvania, Unites States).

### Permeability measurement

HULEC-5a cells (3 ×10^5^ cells/well) were seeded on a 24-hole polyester transfer hole (Costar) with a 0.4 mm pore size for 24 h. The cells were then infected with the influenza virus, MOI 0.01. Endothelial permeability was evaluated by measuring trans-endothelial electrical resistance (TEER) at 0, 8, 16, 24, and 48 h after stimulation using the EVOM2 Epithelial Voltohmmeter (Bio-Rad, Hercules, CA, USA). Untreated HULEC-5a cells were used as a negative control, and medium alone was used as a blank control. Relative TEER was expressed as follows: [(resistance in the experimental group) - (resistance in medium alone)]/[(resistance in untreated HUVECs)-(resistance in medium alone)]; resistance was expressed in ohms.

### Target gene for pulmonary microvascular leakage caused by H1N1

We performed text mining in the GeneCards database (version 5.0) (https://www.genecards.org/Search/Keyword?queryString=Pulmonary%20microvascular%20lea kage%20caused%20by%20H1N1). The GeneCards is a searchable, integrative database that provides comprehensive, user-friendly information on all annotated and predicted human genes. The knowledgebase automatically integrates gene-centric data from 150 web sources, including genomic, transcriptomic, proteomic, genetic, clinical, and functional information. The keyword was “Pulmonary microvascular leakage caused by H1N1”. All targets were restricted to human origin.

### Functional and signal pathway enrichment analysis

To systematically understand the biological processes of the H1N1 virus causing pulmonary microvascular leakage, we conducted enrichment analysis of potential targets using gene ontology (GO) and Kyoto Encyclopedia of Genes and Genomes (KEGG). DAVID Bioinformatics Resources (version 6.8) provides a comprehensive set of functional annotation tools for investigators to understand the biological meaning behind a large list of genes. DAVID (https://david.ncifcrf.gov/tools.jsp) was used to perform GO analysis, including biological process (BP), cellular component (CC), molecular function (MF), and KEGG Pathway analysis. The terms with a *p*-value of less than 0.05 were selected for functional annotation and signaling pathway clustering (Huang, Sherman & Lempicki, 2009a; Huang, Sherman & Lempicki, 2009b; Lai et al., 2021).

### Protein-Protein interaction (PPI) analysis and gene module analysis

The STRING search tool was used to provide a key assessment of the protein-protein functional associations, with the species limited to “Homo sapiens.” The protein-protein interaction network (PPI) among the potential targets was constructed using the STRING search tool (version 11.0) (https://www.string-db.org/cgi/input?sessionId=bvSr4bpeU0nK&input_page_active_form=multiple_sequences), and the dates were saved as “CSV”. Cytoscape app was used for subsequent analysis. The molecular complex detection (MCODE) algorithm, was used to discover the closely related areas in the PPI network, by calculating the node information of each node in the network. The dates were run on the MCODE and STRING modules in Cytoscape, and the top one protein interaction module was saved for the next analysis (Halary et al., 2010).

### Drug-gene interaction analysis

DGIdb database (version 3.0) (https://dgidb.org/search_interactions) was used to search for potential targets with existing associations with existing drugs or small organic compounds. We selected the genes of the top one protein interaction module and click “Find Drug-Gene Interactions” to identify the drugs. Here, we set the preset filter to all approved drugs (Cotto et al., 2018).

## Results

### H1NI virus infects human lung microvascular endothelium

**Figure 1:**
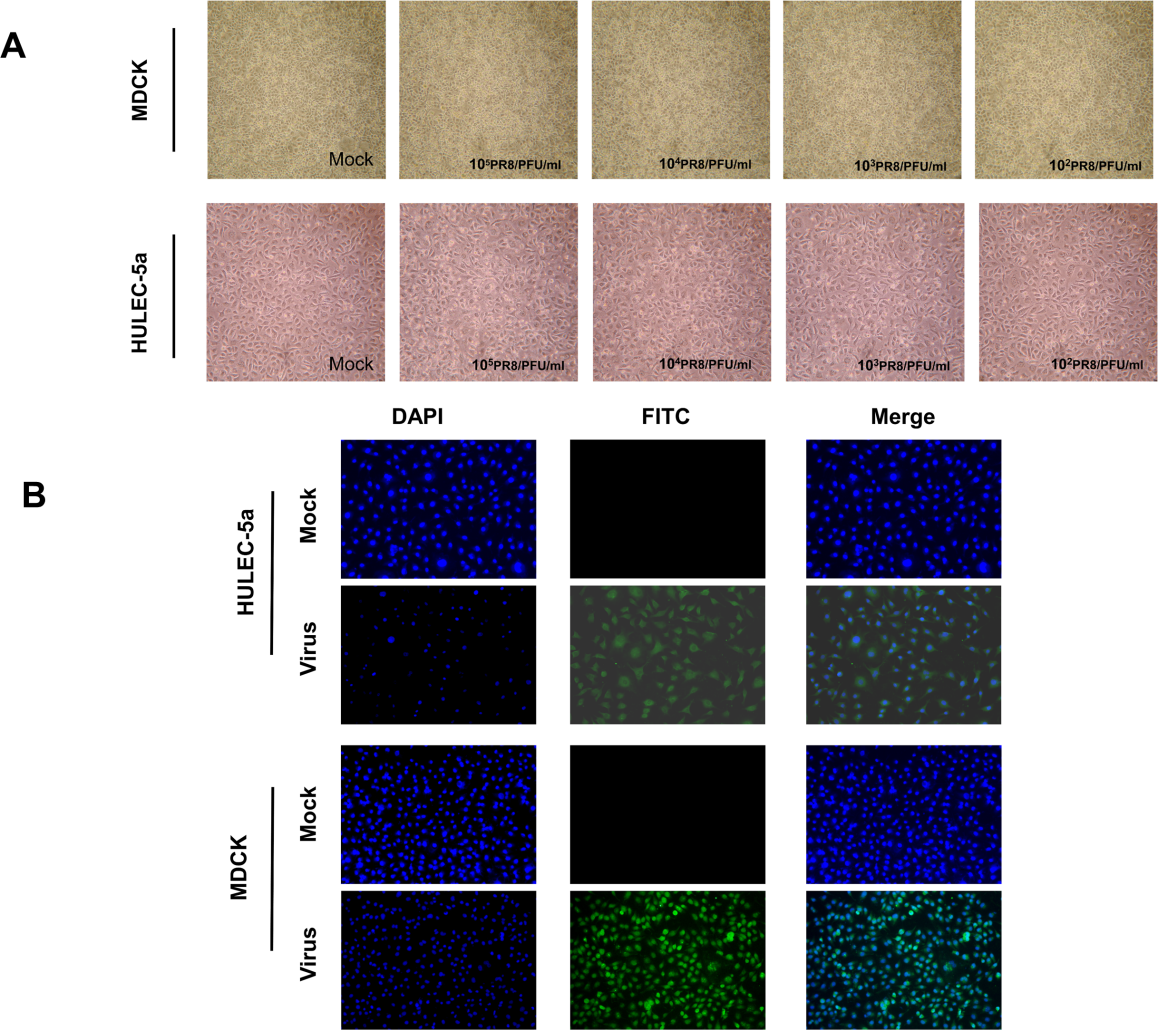
Influenza virus can infect endothelial cells, but cannot cause obvious cytopathic changes. (A) 48 h after infection of MDCK cells and HULEC-5a with influenza virus of different dilutions, pathological changes were observed under the microscope. (B) 48 h after infection of MDCK cells and HULEC-5a with influenza virus of MOI 0.01. The cells were fixed and immunostained with nucleoprotein (green). Counterstaining was performed using DAPI (blue).

**Figure 2:**
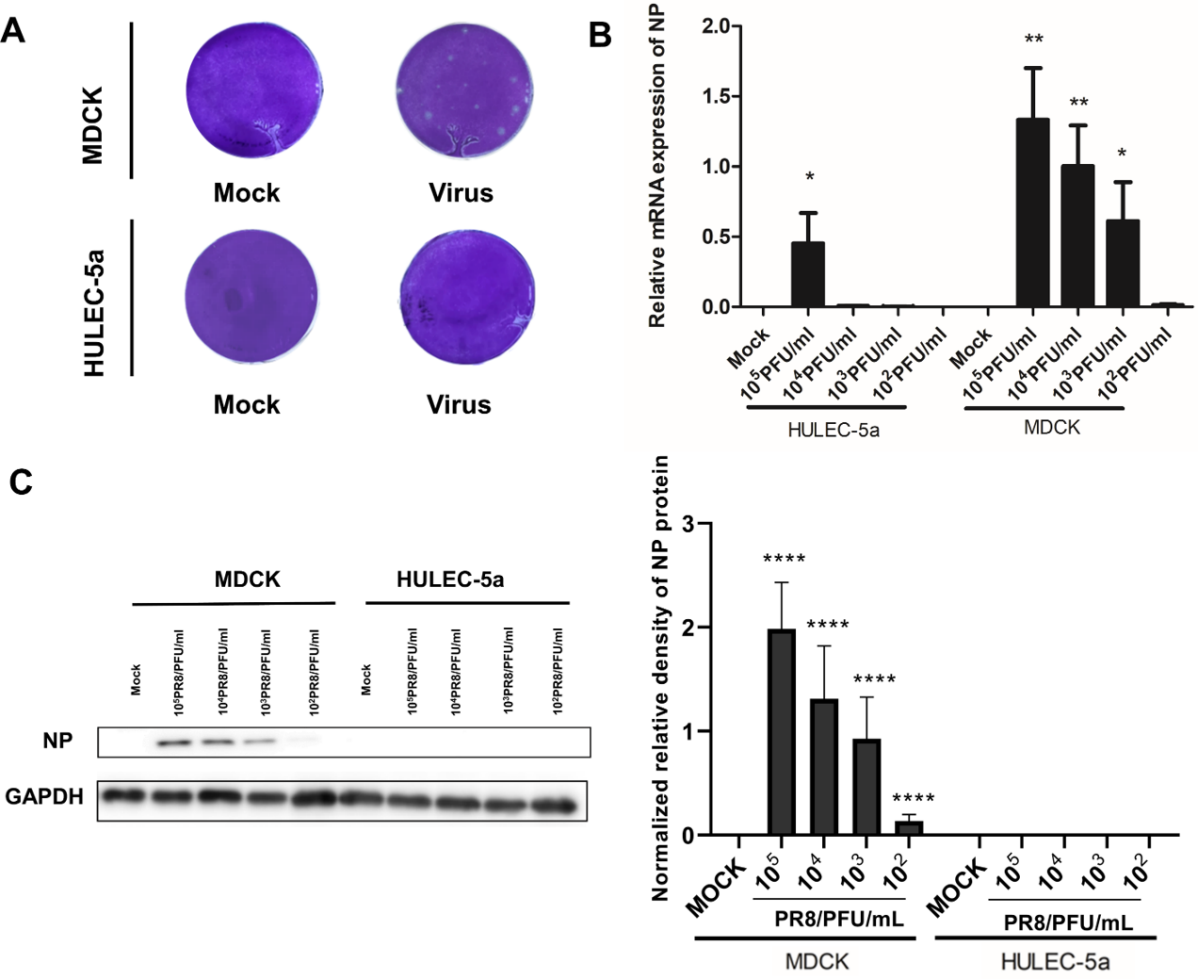
Influenza virus can initiate RNA replication after infecting endothelial cells, but cannot initiate protein replication, and cannot produce progeny viruses. (A) 48 h after infection, the virus titer in MDCK cells infected with influenza virus and HULEC-5a supernatant was measured by the plaque formation method. The dilution factor of the virus group in the figure is 10^5^-fold and the results represent three experiments. (B) After infection with influenza virus of different dilutions, real-time quantitative PCR analysis using nucleoprotein specific primers confirmed that the virus can replicate on endothelial cells and MDCK, *p < 0.05. The results are representative of three experiments. (C) After influenza virus infection, HULEC-5a cells and MDCK cells were incubated for 48 h, and the level of NP protein was determined by WB analysis, as described in the “Materials and methods” section. The gray level of each band was measured by Image J software to detect the difference of NP protein expression. Data are mean ± SD of three experiments. Compared with the control group, *p < 0.05. The picture shows the representative data of three independent experiments.

MDCK and HULEC-5a cells were infected with different dilutions of the A/PR/8/34 (H1N1) influenza virus for 48 h. Pathological changes of endothelial cells under the microscope were not obvious (Fig. 1A). Immunofluorescence staining results showed that A/PR/8/34 (H1N1) influenza virus-infected both MDCK and HULEC-5a cells (Fig. 1B). The supernatants collected from MDCK and HULEC-5a cells were used to detect the virus titer. The results showed that the virus titer for the MDCK cells was 1.9*10^5^ PFU/ml, while that for the endothelial cell was 0 PFU/ml (Fig. 2A). We determined the replication of A/PR/8/34 (H1N1) influenza virus nucleoprotein (NP) mRNA in endothelial cells by real-time quantitative PCR. The results showed that endothelial cells infected with the virus (MOI 0.00001, 0.0001, 0.0001, 0.001, 0.01) had increased NP mRNA (Fig. 2B). Finally, western blotting was used to detect the expression of viral nucleoprotein. At different virus titers, endothelial cells did not produce NP protein within 48 h (Fig. 2C, Supplement File S1, File S2). A/PR/8/34 (H1N1) influenza virus was found to infect HULEC-5a cells and initiate viral gene transcription but did not express a viral protein or produce infectious offspring.

### Viral infection of microvascular endothelial cells leads to increased permeability

**Figure 3:**
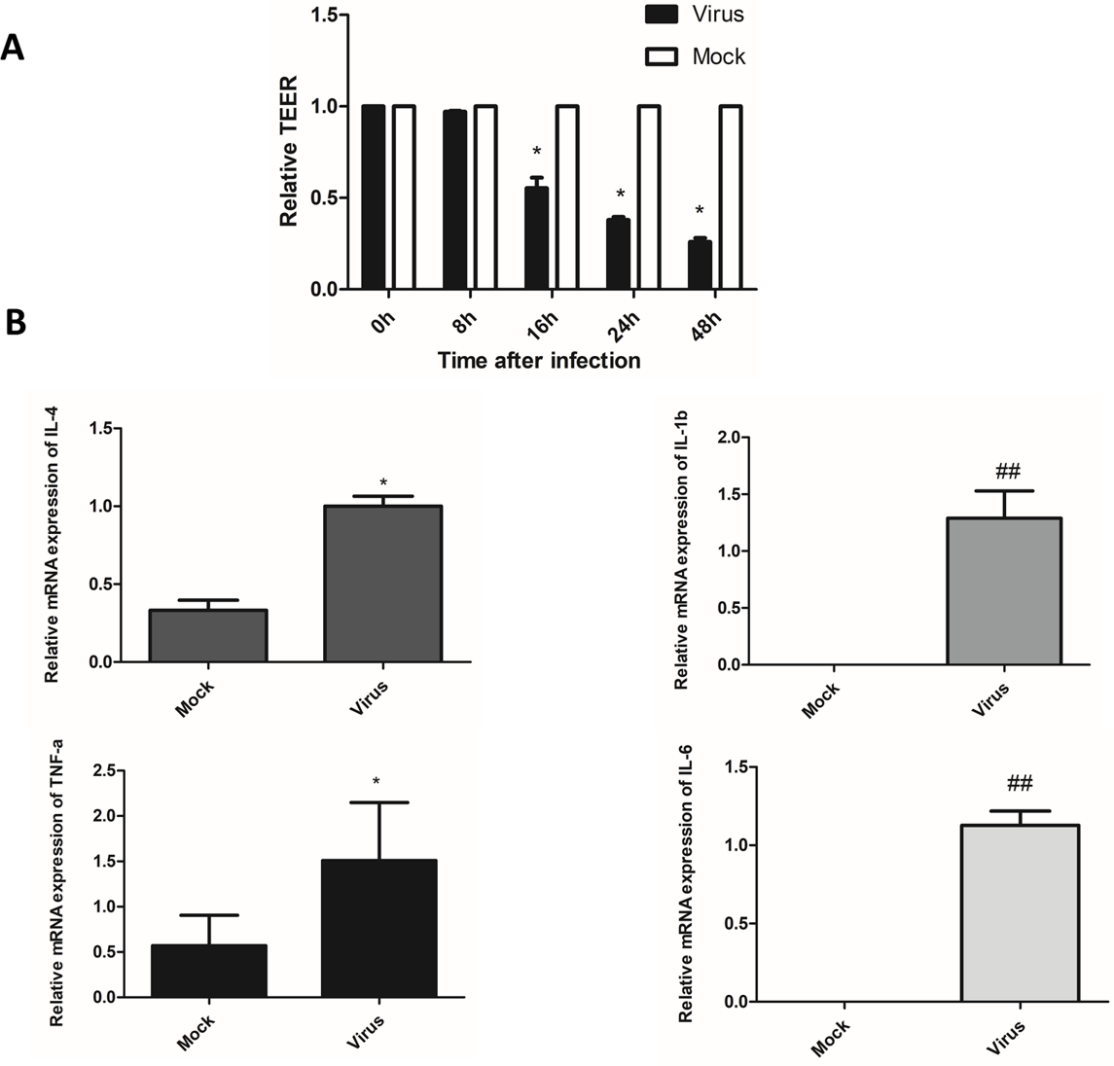
After influenza virus infects endothelial cells, it can cause vascular leakage and induce endothelial cells to produce pro-inflammatory factors. (A) After being infected with influenza virus (MOI 0.01), HULEC-5a cells were incubated for 0, 8, 16, 24 and 48 h. Then the trans-endothelial resistance is detected. Results (mean, SE) are from 3 experiments and are normalized to control, *p < 0.05. B: After infected with influenza virus (MOI 0.01), HULEC-5a cells were incubated for 48 h, and the mRNA level of TNF-α, IL-1β, IL-4, IL-6 was detected by real-time quantitative PCR. Data are mean ± SD of three experiments. *p < 0.05, ^##^*p* < 0.01 versus control group.

The fused HULEC-5a cells formed a compact monolayer on the semipermeable membrane, and the initial resistance value was measured before the cells were infected with the virus. Afterward, the old medium in the upper and lower chambers was removed, and influenza virus was added to the upper chamber at a multiplicity of infection (MOI) of 0.01, and maintenance solution was added to the lower chamber. Two hours after infection with the virus, the liquid in the upper and lower chambers was withdrawn, rinsed with PBS, and maintenance liquid added to both the upper and lower chambers. Resistance was determined at 0, 8, 16, 24, and 48 h after virus removal. A decrease in TEER was associated with increased permeability, while increased TEER suggested enhanced integrity of HULEC-5a cells (Fig. 3A). We observed that the TEER level significantly dropped at 16 h after influenza virus infection, and was at its lowest at 48 h. This indicated that viral infection increased the permeability of lung endothelial cells.

### H1N1 virus induces the production of inflammatory cytokines

Cytokines are considered to be useful indicators of the severity of influenza infection and lung pathological characteristics, and endothelial cells are at the center of cytokine expansion during influenza virus infection (Teijaro et al., 2011). Real-time quantitative PCR was used to detect the mRNA relative expression of pro-inflammatory cytokines, including TNF-α, IL-1β, IL-4, and IL-6 in the supernatant of endothelial cells 48 h after infection with influenza virus. The results showed that the relative mRNA expression levels of TNF-α, IL-1β, IL-4, and IL-6 were significantly higher than that of the control cells (Fig. 3B). The levels of inflammatory cytokines TNF-α, IL-1β, IL-4, and IL-6 were significantly higher than those in the control cells (Fig. 3B). This indicated that endothelial cells could induce the production of inflammatory cytokines after infection with the influenza virus.

### Target gene for pulmonary microvascular leakage caused by H1N1

The GeneCards database was used to perform text mining, and search for genes related to “pulmonary microvascular leakage caused by H1N1”, Through text mining, a total of 107 genes related to pulmonary microvascular leakage caused by H1N1 (Table 1, Supplement File S3) were identified. This gene set was saved for the next analysis.

**Table 1:** A total of 107 genes related to pulmonary microvascular leakage caused by H1N1.

Gene symbol	Description	Relevance score
TNF	Tumor Necrosis Factor	74.35
ALB	Albumin	51.15
VEGFA	Vascular Endothelial Growth Factor A	47.27
IL6	Interleukin 6	39.39
IL1B	Interleukin 1 Beta	34.93
ICAM1	Intercellular Adhesion Molecule 1	33.81
TGFB1	Transforming Growth Factor Beta 1	32.16
CCL2	C-C Motif Chemokine Ligand 2	28.96
INS	Insulin	28.92
F2	Coagulation Factor II, Thrombin	27.45
ELANE	Elastase, Neutrophil Expressed	24.86
IL10	Interleukin 10	23.96
SFTPB	Surfactant Protein B	23.72
CXCL8	C-X-C Motif Chemokine Ligand 8	23.26
CRP	C-Reactive Protein	22.93
SOD1	Superoxide Dismutase 1	22.64
IFNG	Interferon Gamma	21.96
MMP2	Matrix Metallopeptidase 2	21.52
NOS2	Nitric Oxide Synthase 2	20.36
HIF1A	Hypoxia Inducible Factor 1 Subunit Alpha	20.23
CRYAA	Crystallin Alpha A	20.11
TLR4	Toll Like Receptor 4	19.37
SOD2	Superoxide Dismutase 2	19.36
FGF2	Fibroblast Growth Factor 2	18.59
PTPN11	Protein Tyrosine Phosphatase Non-Receptor Type 11	18.59
TP53	Tumor Protein P53	18.39
CCR5	C-C Motif Chemokine Receptor 5	18.29
MMP9	Matrix Metallopeptidase 9	18.08
MBL2	Mannose Binding Lectin 2	17.33
TLR2	Toll Like Receptor 2	16.61
IFNA1	Interferon Alpha 1	16.13
NEU1	Neuraminidase 1	15.85
FN1	Fibronectin 1	14.34
IFNB1	Interferon Beta 1	14.04
CCL5	C-C Motif Chemokine Ligand 5	13.84
EGFR	Epidermal Growth Factor Receptor	13.73
B2M	Beta-2-Microglobulin	13.07
TLR3	Toll Like Receptor 3	12.45
KNG1	Kininogen 1	12.13
MIR155	MicroRNA 155	12.11
SRC	SRC Proto-Oncogene, Non-Receptor Tyrosine Kinase	11.72
AGER	Advanced Glycosylation End-Product Specific Receptor	11.58
CD55	CD55 Molecule (Cromer Blood Group)	11.23
TTR	Transthyretin	10.91
CD209	CD209 Molecule	10.75
CXCL10	C-X-C Motif Chemokine Ligand 10	10.49
IL1A	Interleukin 1 Alpha	10.25
CASP3	Caspase 3	10.2
APP	Amyloid Beta Precursor Protein	9.96
IL5	Interleukin 5	9.69
CSF2	Colony Stimulating Factor 2	9.63
IL2	Interleukin 2	9.58
CALCA	Calcitonin Related Polypeptide Alpha	9.55
PTGS2	Prostaglandin-Endoperoxide Synthase 2	9.45
NFKB1	Nuclear Factor Kappa B Subunit 1	9.32
IL18	Interleukin 18	9.2
CCL4	C-C Motif Chemokine Ligand 4	9.2
NFE2L2	Nuclear Factor, Erythroid 2 Like 2	8.78
CXCR3	C-X-C Motif Chemokine Receptor 3	8.4
BAX	BCL2 Associated X, Apoptosis Regulator	8.39
MAPK14	Mitogen-Activated Protein Kinase 14	8.31
MIR146A	MicroRNA 146a	8.2
PLAU	Plasminogen Activator, Urokinase	8.17
TLR5	Toll Like Receptor 5	8.15
HMGB1	High Mobility Group Box 1	7.69
BCL2	BCL2 Apoptosis Regulator	7.44
IFNGR1	Interferon Gamma Receptor 1	7.28
ABCB1	ATP Binding Cassette Subfamily B Member 1	7.03
RELA	RELA Proto-Oncogene, NF-KB Subunit	6.93
RHOA	Ras Homolog Family Member A	6.69
JUN	Jun Proto-Oncogene, AP-1 Transcription Factor Subunit	6.43
PIK3CG	Phosphatidylinositol-4,5-Bisphosphate 3-Kinase Catalytic Subunit Gamma	6.4
IL2RA	Interleukin 2 Receptor Subunit Alpha	6.28
IL3	Interleukin 3	5.97
MIF	Macrophage Migration Inhibitory Factor	5.95
IL9	Interleukin 9	5.89
PRKCD	Protein Kinase C Delta	5.88
ACTN4	Actinin Alpha 4	4.95
GHRL	Ghrelin And Obestatin Prepropeptide	4.92
PTK2	Protein Tyrosine Kinase 2	4.84
IFITM3	Interferon Induced Transmembrane Protein 3	4.79
HSPD1	Heat Shock Protein Family D (Hsp60) Member 1	4.72
MRC1	Mannose Receptor C-Type 1	4.59
F2RL1	F2R Like Trypsin Receptor 1	4.48
AQP1	Aquaporin 1 (Colton Blood Group)	4.36
IRF3	Interferon Regulatory Factor 3	4.24
HSPA1A	Heat Shock Protein Family A (Hsp70) Member 1A	4.23
PLCG1	Phospholipase C Gamma 1	4.02
CAMP	Cathelicidin Antimicrobial Peptide	3.99
AQP5	Aquaporin 5	3.76
EZR	Ezrin	3.73
PTPA	Protein Phosphatase 2 Phosphatase Activator	3.42
XBP1	X-Box Binding Protein 1	3.25
KLF2	Kruppel Like Factor 2	3.18
GLUL	Glutamate-Ammonia Ligase	3.16
PIK3CB	Phosphatidylinositol-4,5-Bisphosphate 3-Kinase Catalytic Subunit Beta	3.14
FYN	FYN Proto-Oncogene, Src Family Tyrosine Kinase	2.88
CXCL11	C-X-C Motif Chemokine Ligand 11	2.65
ISG15	ISG15 Ubiquitin Like Modifier	2.6
SGPL1	Sphingosine-1-Phosphate Lyase 1	2.6
ITGA5	Integrin Subunit Alpha 5	2.52
AIFM1	Apoptosis Inducing Factor Mitochondria Associated 1	2.4
SIGMAR1	Sigma Non-Opioid Intracellular Receptor 1	2.07
CLEC4A	C-Type Lectin Domain Family 4 Member A	1.65
DEFA1	Defensin Alpha 1	1.54
RIPK3	Receptor Interacting Serine/Threonine Kinase 3	1.52
PTPRB	Protein Tyrosine Phosphatase Receptor Type B	1.11

### Functional and signal pathway enrichment analysis

**Figure 4:**
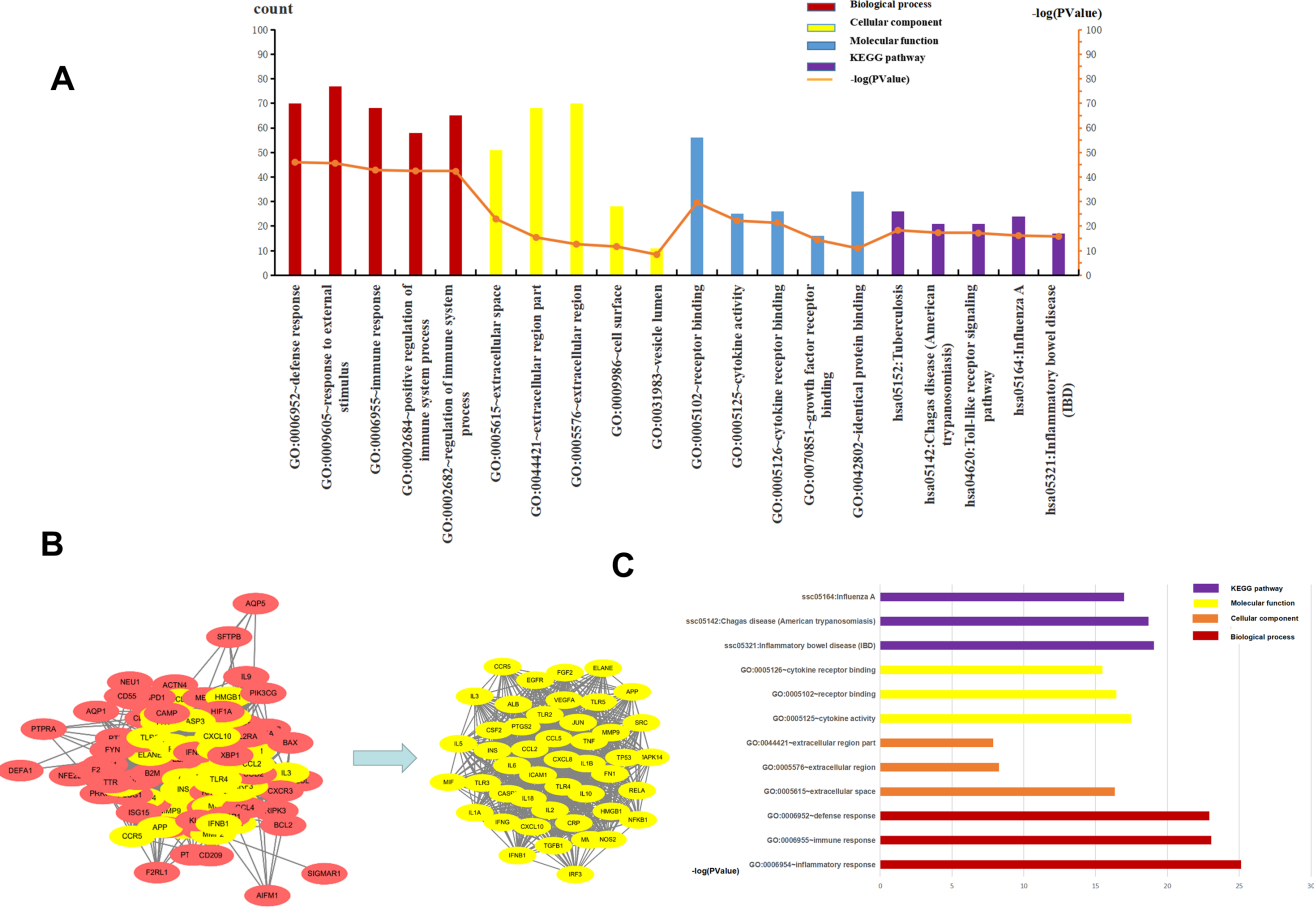
The top five of GO enrichment and KEGG pathway enrichment and PPI network. The top three of GO enrichment and KEGG pathway enrichment of hub genes. (A) The top five of GO enrichment and KEGG pathway enrichment. (B) PPI network. the 107 common genes and network of hub genes. (C) The top three of GO enrichment and KEGG pathway enrichment of hub genes.

The top five BP, CC, MF, and KEGG pathways were determined using DAVID. For CC, the genes were mainly enriched in the extracellular space, extracellular region part, cell surface, and vesicle lumen (Supplement File S4). For BP the genes were closely related to “defense response”, “response to external stimulus”, “immune response”, “positive regulation of immune system process” and “regulation of immune system process” (Supplement File S5). The results of MF enrichment showed that they were primarily associated with “receptor binding”, “cytokine activity”, “cytokine receptor binding”, “growth factor receptor binding” and “identical protein binding” (Supplement File S6). The top five highly enriched Signal Pathways were “Tuberculosis”, “Chagas disease (American trypanosomiasis)”, “Toll-like receptor signaling pathway”, “Influenza A” and “Inflammatory bowel disease (IBD)” (Supplement File S7, Supplement File S8, Fig. 4A). These results showed that BP was mainly related to immune response, CC was mainly related to the extracellular region and vesicle lumen, MF was mainly related to cytokines, and KEGG Pathway was mainly related to inflammation signaling pathway and immune signaling pathway.

### PPI and gene module analysis

To understand the relationship between the targets more intuitively, 107 targets were imported into the SRING database to construct a gene functional association network, and the minimum score of protein interaction was set to 0.400. The network contained 105 nodes, 1808 edges, and a network graph with an average node degree of 34.4. The nodes represented the target protein, and the edges represented the protein-protein interaction relationship. STRING was used to analyze the protein-protein interaction of the genes using a protein interaction network diagram. We saved the file in “TSV” format and used Cytoscape to analyze the file (Supplement 9). The levels of TNF, IL6, TLR4, VEGFR, ALB, IL-10, IL-1β, and CXCL8 were significantly higher than other targets that play an important role in the PPI network. The highest target in this network was TNF, with a degree value of 82, which may be the most important potential target for H1N1 in causing pulmonary microvascular leakage. We used the MCODE plug-in in Cytoscape to perform module analysis on the file and obtained 39.111 points, ranking first, cluster-1 with 46 core genes (Supplement File S10, Fig. 4B). Further analysis of cluster-1 showed that the first five genes TNF, IL6, TLR4, IL-1β, and IL-10 had the highest degree values, and play a very important role in the cluster-1 network.

### Functional and signal pathway enrichment analysis of module

Cluster-1 was further analyzed by GO and KEGG and based on the *p*-value, the first three BP, CC, MF, and KEGG were identified. CC enrichment was mainly enriched in the extracellular space and the extracellular area. For BP the enrichment was closely related to “defense response”, “inflammatory response” and “immune response”. For MF, the enrichment was mainly related to “receptor binding”, “cytokine activity” and “cell Factor receptor binding”. The top 3 most enriched signal pathways were “Chagas disease (American trypanosomiasis), ”Influenza A”’ and “Inflammatory bowel disease (IBD)” (Fig. 4C). In summary, BP was mainly related to the immune response, CC to the extracellular area, MF to cytokines, and KEGG pathway analysis was mainly related to inflammatory signaling pathway and immune signaling pathway. H1N1 was found to mainly cause pulmonary microvascular leakage in the extracellular area, activate and produce cytokines, and cause inflammation and immune responses. The signal pathway was mainly related to the inflammatory signaling pathway and the immune signaling pathway.

### Drug-gene interaction analysis

The DGIdb tool was used to search and screen 46 genes in cluster-1 for drug-gene interaction analysis (Supplement File S11). As shown in Table 2, a list of approved drugs that meet the requirements of treatment standards has been compiled. There were 42 potential genetic targets in this list, and the corresponding drugs were ranked first in the Interaction Score & Query Score. Common uses of these drugs included cancer treatment, anti-inflammation, vascular disease treatment, immune function regulation, and hormone regulation.

**Table 2:** Drug acting on 46 hub gene.

Gene	Drug	Interaction_types	PMID
IFNG	GLUCOSAMINE		16155294
CCL2	DANAZOL	inhibitor	11056242
MAPK14	DOXORUBICIN		16905201
NFKB1	ARTESUNATE		25074847
IL3	AMLEXANOX	antagonist	1378042
IL5	MEPOLIZUMAB	antagonist	18564273
CRP	ADALIMUMAB		27096233
IL2	IMIPENEM		7796708
TLR2	ADAPALENE	antagonist	26947815
FGF2	SUCRALFATE	inducer	7948825
IL1B	CANAKINUMAB	inhibitor	19169963
IL1A	RILONACEPT	binder	23319019
TP53	CISPLATIN		25376608
ELANE	FILGRASTIM	other/unknown	15353486
ICAM1	LIFITEGRAST		
EGFR	AFATINIB	inhibitor	26619011
ALB	OLMESARTAN MEDOXOMIL		22086979
IL18	TACROLIMUS		25712187
CASP3	MINOCYCLINE	negative modulator	12112047
CSF2	TICARCILLIN		7875147
PTGS2	ETORICOXIB	inhibitor	17573128
TLR3	HYDROXYCHLOROQUINE		16142732
JUN	CUPRIC CHLORIDE		
CXCL8	TALC		17000556
SRC	DASATINIB	multitarget	27231123
CCR5	MARAVIROC	antagonist	16298345
HMGB1	ITRACONAZOLE		14639950
TLR4	MIFAMURTIDE	ligand	21226638
MMP9	GLUCOSAMINE	antagonist	12405690
INS	INULIN		
IL6	SILTUXIMAB	antagonist	8823310
IL10	RITUXIMAB		26384320
TNF	INFLIXIMAB	inhibitor	16720636
VEGFA	RANIBIZUMAB	inhibitor	18046235
TGFB1	HYALURONIDASE	inhibitor	9435505
TLR5	USTEKINUMAB		28696418
NOS2	ARGININE		16778961
RELA	ARTESUNATE		25074847
APP	TROMETHAMINE	inhibitor	8380642
FN1	ZINC CHLORIDE	modulator	23896426
CXCL10	METHYLPREDNISOLONE		17220550
MMP2	CAPTOPRIL	inhibitor	17308006

## Discussion

Influenza A virus lacks a complete enzymatic system and has no raw materials and energy to synthesize its components. Therefore, the virus must invade susceptible host cells and rely on the host cell’s enzymatic system, raw materials, and energy to complete replication and produce progeny viruses. The process of influenza A virus replication is divided into five steps: Adsorption, penetration, uncoating, biosynthesis, assembly, and release, also known as the replication cycle (Steinberg, Goldenberg & Lee, 2012). Influenza A virus can infect endothelial cells (Fig. 1B), however, no obvious lesions are seen under the microscope (Fig. 1A). The plaque assay results showed that no offspring of the influenza A virus was produced (Fig. 2A). Further detection of the relative expression level of NP mRNA (Fig. 2B) and NP protein expression level (Fig. 2C), showed that the virus could replicate NP mRNA in endothelial cells, but could not synthesize the corresponding NP protein. This indicated that the influenza A virus did not complete the replication cycle after infecting endothelial cells.

We also studied the permeability of virus-infected endothelial cells and cytokine production. The permeability of endothelial cells was found to increase after influenza virus infection (Fig. 3A), and the levels of pro-inflammatory factors such as IL-1β, TNF-α, IL-4, and IL-6 also increased (Fig. 3B). Studies have confirmed that IL-1β and TNF-α can increases endothelial permeability (Ferro et al., 2000; Kim et al., 2015). In this study, we speculated that viral infection of endothelial cells could cause an increase in the level of pro-inflammatory factors, which affect the permeability of endothelial cells. We also speculated that the production of large amounts of inflammatory cytokines resulted in endothelial cell damage.

To further understand the mechanism of vascular leakage caused by the influenza A virus, we used bioinformatics tools to screen for the genes related to H1N1-induced pulmonary microvascular leakage, explore the potential molecular pathways and understand protein-protein interaction relationships. The results showed that 107 potential targets and biological processes of core genes were mostly related to inflammation and immunity. The CC enrichment results were mainly related to the extracellular region and vesicle lumen, the BP enrichment results were mainly related to immune response, and the MF enrichment results were primarily associated with “receptor binding”, “cytokine activity”, “cytokine receptor binding”, “growth factor receptor binding” and “identical protein binding”. The KEGG pathway enrichment was mainly involved in pathways such as “inflammatory bowel disease” (IBD), “Chagas disease” (Chagas disease), and “influenza A”, suggesting that the reason why influenza virus causes increased permeability of vascular endothelial cells may be related to the activation of these pathways.

Combining the current literature and the results of this study, the possible reasons for the influenza virus to increase the permeability of vascular endothelial cells are as follows: (1) The influenza virus can infect endothelial cells, activate signaling pathways, and induce endothelial cell apoptosis, thereby destroying the epithelial-endothelial barrier. The signaling pathway associated with increased vascular endothelial permeability caused by the influenza virus may be the signaling pathway enriched by KEGG, but this still needs further experimental verification. (2) The influenza virus can induce endothelial cells to produce inflammatory cytokines, aggravate the cytokine storm, and increase damage to endothelial cells. Teijaro et al. (2011) showed that endothelial cells were the central regulators of cytokine expression during influenza viral infection. Endothelial cells are potential targets for suppressing excessive innate inflammation. (3) The CC enrichment results were mainly related to the extracellular region and vesicle lumen. This suggests that the reason for the increase in endothelial cell permeability caused by the influenza virus may be related to the disintegration of the connections between endothelial cells or the secretion of exosomes from peripheral cells to affect endothelial cell permeability.

These results indicate that, after infection with the A/Puerto Rico/8/34 (H1N1) influenza virus, endothelial cells could not effectively replicate to produce progeny virions, but caused vascular leakage and cytokine production and release. This finding can exclude the direct impact of the virus on endothelial cells and be used to study other mechanisms that cause increased endothelial cell permeability.

In addition, the currently studied drugs for enhancing vascular barrier, such as Slit2N, can enhance the adhesion of VE-cadherin at the endothelial cell–cell junction in *vitro* without changing the level of the virus titer (London et al., 2010); Doxycycline reduces vascular leakage by inducing the expression of VE-cadherin, the main component of cell adhesion (Ng et al., 2012); Vasculotide is a synthetic Tie2-agonist, which has been shown to reduce the loss of VE-cadherin and cytoskeleton rearrangement (Sugiyama et al., 2015); There are other barrier-enhancing agents, such as atrial natural peptide and sphingosine-1-phosphate, that have been tested in animals in various vascular leakage models (Jiang et al., 2017). Most of the reported drugs act on the junctions between cells. In this study, the DGIdb tool was used to predict the approved drugs on the market, which can also be used to treat other diseases, including cancer, inflammatory diseases, immune system disorders, and cardiovascular diseases. This can provide new clues for drug-related research for the prevention and treatment of vascular leakage, following influenza A virus infection.

## Conclusions

In summary, pulmonary endothelium plays an important role in the pathogenesis of severe influenza. The study of the replication kinetics and host response of the influenza A virus is of great significance for pathogenesis and treatment. This study findings reveal that enhancing the integrity of the endothelial barrier is an attractive treatment option, since it does not directly target the virus, and it is less vulnerable to viral mutations. Therefore, in clinical practice, vascular barrier enhancers should be used in combination with antiviral drugs.

##  Supplemental Information

10.7717/peerj.11892/supp-1 Supplemental Information 1 Western Blot of NP protein

10.7717/peerj.11892/supp-2 Supplemental Information 2 Western Blot of GAPDH protein

10.7717/peerj.11892/supp-3 Supplemental Information 3 Pulmonary microvascular leakage caused by H1N1 related genes from GeneCards

10.7717/peerj.11892/supp-4 Supplemental Information 4 Cellular component of Pulmonary microvascular leakage caused by H1N1 related genes

10.7717/peerj.11892/supp-5 Supplemental Information 5 Biological process of Pulmonary microvascular leakage caused by H1N1 related genes

10.7717/peerj.11892/supp-6 Supplemental Information 6 Molecular function of Pulmonary microvascular leakage caused by H1N1 related genes

10.7717/peerj.11892/supp-7 Supplemental Information 7 KEGG Pathway analysis of Pulmonary microvascular leakage caused by H1N1 related genes

10.7717/peerj.11892/supp-8 Supplemental Information 8 The top 5 gene hits included in the GO enrichment in Fig. 4A

10.7717/peerj.11892/supp-9 Supplemental Information 9 Protein-Protein interaction analysis by STRING

10.7717/peerj.11892/supp-10 Supplemental Information 10 Hub genes after clustering analyzed

10.7717/peerj.11892/supp-11 Supplemental Information 11 Drug acting on 47 hub gene

10.7717/peerj.11892/supp-12 Supplemental Information 12 Relative mRNA expression of IL-1b

10.7717/peerj.11892/supp-13 Supplemental Information 13 Relative TEER

10.7717/peerj.11892/supp-14 Supplemental Information 14 Relative mRNA expression of TNF-a

10.7717/peerj.11892/supp-15 Supplemental Information 15 Western blot of NP and GAPDH

10.7717/peerj.11892/supp-16 Supplemental Information 16 MRNA OF NP protein

## Abbreviations

WB: Western blot
GO: Gene ontology
KEGG: Kyoto Encyclopedia of Genes and Genomes
PPI: Protein-Protein interaction
MDCK: Madin-Darby canine kidney
HULEC-5a: Human lung microvascular endothelial cell line
NP: Nucleoprotein
BP: Biological process
CC: Cellular component
MF: Molecular function

## Additional Information and Declarations

### Data Availability

The following information was supplied regarding data availability: The raw data is available in the Supplementary Files.
