# Supplementary figures and images for: Influenza A virus infects pulmonary microvascular endothelial cells leading to microvascular leakage and release of pro-inflammatory cytokines
Source: PeerJ. 2021 Aug 3;9:e11892. doi: 10.7717/peerj.11892 (PMC8344683; doi:10.7717/peerj.11892)

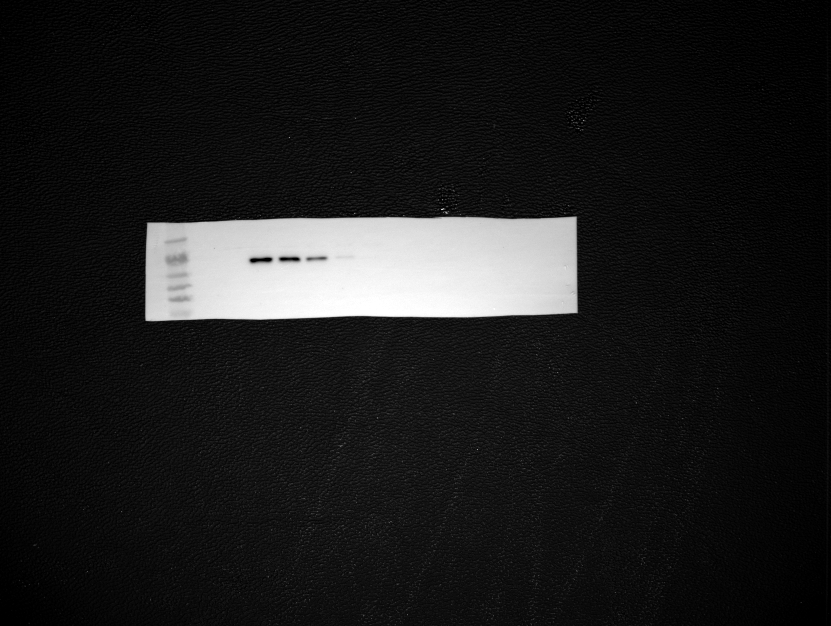

Supplement: Supplemental Information 1 [file peerj-09-11892-s001.png]

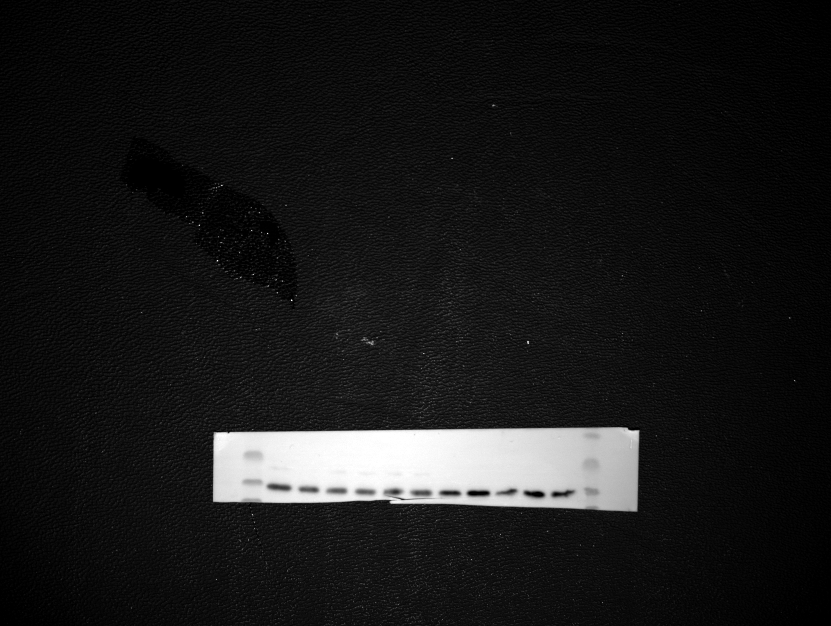

Supplement: Supplemental Information 2 [file peerj-09-11892-s002.png]
